# Thyroid autoimmunity and function among Ugandan children and adolescents with type-1 diabetes mellitus

**DOI:** 10.11604/pamj.2014.19.137.5115

**Published:** 2014-10-09

**Authors:** Rugambwa Michael Muhame, Edison Arwanire Mworozi, Karen McAssey, Irene Lubega

**Affiliations:** 1 Department of Paediatrics and Child Health, School of Medicine, Makerere University College of Health Sciences, Kampala, Uganda; 2 Department of Paediatrics, Mulago National Referral Hospital, Kampala, Uganda; 3 McMaster Children's Hospital/McMaster University, Hamilton, Ontario, Canada

**Keywords:** Thyroid, autoimmunity, type 1 diabetes mellitus, children

## Abstract

**Introduction:**

Up to 30% of type-1 diabetes mellitus (T1DM) patients have co-existent thyroid autoimmunity with up to 50% of them having associated thyroid dysfunction. Routine screening for thyroid autoimmunity and dysfunction is recommended in all T1DM patients. However, this was not currently practiced in Ugandan paediatric diabetes clinics. There was also paucity of data regarding thyroid autoimmunity and dysfunction in African children and adolescents with diabetes mellitus. The objective of this study was to quantify the magnitude of thyroid autoimmunity and dysfunction in Ugandan children with TIDM.

**Methods:**

This was a cross sectional descriptive study to determine the prevalence of thyroid autoantibodies and describe thyroid function among children and adolescents aged 1-19 years with diabetes mellitus attending the paediatric diabetes clinic at Mulago National Referral Hospital, Kampala, Uganda. Following enrollment, we obtained details of clinical history and performed physical examination. Blood (plasma) was assayed to determine levels of antibodies to thyroid peroxidase (antiTPO), free thyroxine (FT4) and thyrotropin (TSH).

**Results:**

The prevalence of thyroid autoimmunity was 7.3% (5/69). All antiTPO positive subjects were post pubertal, aged between 13-17 years with females comprising 3/5 of the antiTPO positive subjects. All study subjects were clinically euthyroid; however, 7.3% (5/69) of the study subjects had subclinical hypothyroidism.

**Conclusion:**

These data strengthen the argument for routine screening of all diabetic children and adolescents for thyroid autoimmunity (particularly anti-TPO) as recommended by international guidelines. We also recommend evaluation of thyroid function in diabetic children and adolescents to minimize the risk of undiagnosed thyroid dysfunction.

## Introduction

Type 1 diabetes mellitus (T1DM) is associated with other immune-mediated disorders such as autoimmune thyroiditis, Addison's disease, pernicious anaemia and celiac disease. [1–3] Up to 30% of patients with T1DM have co-existent thyroid autoimmunity [4–7] and a high prevalence of thyroid dysfunction. [4, 6–9] Thyroid dysfunction predominantly manifests as hypothyroidism in up to 50% of antibody positive subjects [8, 9] with up to 3% presenting with hyperthyroidism.^4^, ^8^] This is in contrast with the general population where up to 3.4% of children and adolescents have thyroid autoantibodies. [10] The presence of thyroid autoantibodies has a high predictivity (up to 50%) for the development of thyroid dysfunction [9]. It is therefore recommended that screening for thyroid autoantibodies and dysfunction should be performed at diabetes mellitus onset or diagnosis in all paediatric patients with T1DM [11, 12] and regular screening is advocated by the International Society of Paediatric and Adolescent Diabetes (ISPAD) Clinical Practice Consensus Guidelines (2009).

Thyroid dysfunction in children and adolescents is known to adversely affect diabetes control, growth, development and overall well-being [8], however, this has not been studied in Ugandan children and adolescents with T1DM. Screening for thyroid dysfunction is not yet part of routine care in Ugandan paediatric diabetes clinics due to resource constraints. In addition, there is limited data on thyroid autoimmunity and dysfunction in African children with T1DM. The only Medline listed study among African children found a prevalence of 8.2% for thyroid autoimmunity [13] however thyroid function was not evaluated in that study. This study therefore aimed to determine the prevalence of thyroid autoantibodies and describe thyroid function among children and adolescents attending the paediatric diabetes clinic at the Mulago National Referral Hospital in Uganda. This study would also provide further documented evidence of the burden of thyroid autoantibodies and thyroid dysfunction among African children and adolescents with T1DM.

## Methods

This study was cross sectional and descriptive and was carried out among children and adolescents with a previous diagnosis of T1DM attending the Paediatric Diabetes Clinic at Mulago National Referral Hospital in Kampala, Uganda, between January and March 2011. Using Daniel's formula [14] for a finite population, taking a standard normal value corresponding to 95% CI and assuming a margin of error of 5% with estimated prevalence of 26%, a sample size of 69 children was calculated from 81 children and adolescents who regularly attended the clinic.

Of the 70 children who attended the clinic during the study period, 69 were enrolled into the study after obtaining written informed consent from the patient caretakers and from patients 18 years and older. In addition, assent was obtained from children 8 years and older. A questionnaire was used to collect clinical information and blood samples were taken. Urine samples were taken from all females aged 8 years and over. Approval for this study was obtained from the Makerere University School of Medicine Research and Ethics Committee and the Uganda National Council for Science and Technology. Levels of antibodies to thyroid peroxidase (Anti-TPO), free thyroxine (fT4) and thyrotropin (TSH) were determined by electrochemiluminescence immunoassay (ECLIA) on the Elecsys 2010 Immunoanalyser (Roche Diagnostics GmbH, Mannheim, Germany). A titre of anti-TPO exceeding 35 IU/ml was considered positive. The normal TSH range was 0.39–4.0 mIU/ml with hypothyroidism considered subclinical for values between 4.1-10 mIU/ml and clinically significant for values >10 mIU/ml. The normal fT4 range was 14–24 pmol/L.

**Statistical Analysis**: Data was entered using EpiData (v3.1) then exported and analysed with STATA (v10). Data was tested for normality using Shapiro Wilks W test and nonparametric data then summarised using proportions, medians and inter quartile ranges. Categorical variables were compared using chi square or Fishers Exact tests and continuous variables were compared using Mann Whitney U tests. A significance level of p < 0.05 was chosen.

## Results

During the study period, 69 children and adolescents with a prior diagnosis of type 1 diabetes mellitus (T1DM) were recruited into the study. The participants were equally distributed by gender with males comprising 50.7% (35/69) of the study group. Other baseline characteristics of the study participants are shown in Table 1

**Table 1:** Characteristics of the study participants

Characteristic	
Sex – Male (n/N (%))	35/69 (50.7)
Age (Yrs) – Median (IQR)	15 (13 – 17)
Median Age at Diagnosis (Yrs) – Median (IQR)	12 (10 – 14)
Duration of Diabetes (Yrs) – Median (IQR)	2.9 (1.3 – 4.4)
Family h/o Thyroid Disease n/N (%)	4/69 (5.8)
Anti TPO antibody Positive n/N (%)	5/69 (7.3)
Abnormal TSH n/N (%)	5/69 (7.3)

A positive family history of diabetes mellitus and thyroid disease were reported in 43.5% (30/69) and 5.8% (4/69) of the study participants, respectively. Eighty two percent (57/69) of the study participants were post pubertal upon evaluation of development using the Tanner staging. In 97% (67/69), the Tanner staging was appropriate for age while 3% (2/69) had delayed puberty. Both of these with delayed puberty were males aged 15 and 18 years respectively with no evidence of pubertal development (Tanner stage 1). In our study, 7.3% (5/69) of the study participants, had clinically significant levels of Anti-TPO >35 IU/ml. Three out of the five antibody positive participants were female, with one of the females being pregnant. Four of these five antibody positive participants had normal thyrotropin (TSH) levels with one having elevated TSH levels. Characteristics of the anti-TPO antibody positive and negative subjects are shown in Table 2

**Table 2:** Characteristics of Anti-TPO antibody positive and negative study participants

Characteristic	Anti-TPO Positive (n= 5)	Anti-TPO Negative (n = 64)	p
Female (N)	3	31	0.5
Male (N)	2	33	
TSH – Normal	4	60	0.3
TSH – Elevated	1	4	
Age (Years) – Median (IQR)	15 (14 – 16)	15 (12 – 17)	0.9
Duration of Diabetes (Years) – Median (IQR)	2.9 (2.8 – 3.8)	2.8 (1.1 – 4.4)	0.5
Age at Diagnosis (Years) – Median (IQR)	12 (12 – 13)	12 (10 – 14)	0.7

All antibody positive study participants were pubertal with Tanner stages between 3-5 for pubarche, thelarche and testicular development. In the antibody negative group, 81.3% (52/64) were pubertal. None of the participants in the antibody positive group had a positive family history of thyroid disease. Regarding thyroid function, all study participants had normal levels of free thyroxine (fT4) levels between 14 – 24 pmol/L. In addition, 92.7% (64/69) of the study participants had normal TSH levels with 7.3% (5/69) having elevated TSH levels. All of those with elevated TSH levels had normal fT4 levels indicating that they had sub-clinical hypothyroidism. Four of the five participants with elevated TSH levels were negative for thyroid peroxidase antibodies with only one participant having both thyroid peroxidase antibodies and thyroid dysfunction.

The study participants with elevated TSH had a median duration of diabetes of 4 (IQR 3.8 - 8.0) years compared to those with normal TSH levels with 2.8 (IQR 1.1 - 4.4) years (p = 0.049). The median height of the subjects with elevated TSH was <3^rd^ percentile (IQR <0.1 – 22.4%) while those with normal TSH had a median height at the 14.3rd (IQR 2.8-39.3) percentile (p = 0.2). However, 34.8% (24/69) of all the study participants were stunted with 60% (3/5) of those with elevated TSH and 32.8% (21/64) of those with normal TSH being stunted (p = 0.4). All the subjects with elevated TSH were post pubertal and none had obvious thyroid enlargement on clinical examination. In addition, they also had elevated HbA1C levels (>7% (53mmol/mol)).

## Discussion

This cross sectional study was done with the aim of establishing the magnitude of thyroid autoimmunity and dysfunction among Ugandan children and adolescents with Type 1 diabetes mellitus. The prevalence of thyroid autoimmunity among children and adolescents with a previous diagnosis of type 1 diabetes mellitus at the Paediatric diabetes clinic of Mulago National Referral Hospital in Uganda was 7.3%; the same as the prevalence of thyroid dysfunction (all study subjects had sub-clinical hypothyroidism). This study provides the first documented evidence of thyroid autoimmunity among Ugandan children and adolescents with T1DM and adds to the literature on the subject in Africa. The findings from this study are similar to the other African study done in Egypt [13] which found a prevalence of 8.2% of thyroid autoimmunity. Compared to the Egyptian study, our study had the additional advantage of having evaluated thyroid function.

The prevalence of thyroid autoimmunity in our study is lower than that reported in other studies outside Africa [4, 5, 8, 15]. This could be due to the T1DM arising mainly by an autoimmune process in the areas where these other studies were done. In African subjects, an idiopathic form of diabetes mellitus has been reported which follows beta cell destruction for which neither an aetiology nor pathogenesis is known [16] which could account for the finding of a lower prevalence of autoimmunity in our study. The prevalence of 7.3% of thyroid autoimmunity in our study is lower than another African study done in Nigeria [17] which found a prevalence of 46% thyroid autoimmunity. However, the latter study was done among adult patients and probably reflects the increasing prevalence of autoimmune disease associated with increasing age [1, 18, 19]. While this study was not powered to explore associations, we found that three of the five antibody positive subjects were female. This is similar to what has been reported in other studies about females being more affected by autoimmune disease than males [6, 8]. This study further found a prevalence of 7.3% of thyroid dysfunction which was all subclinical hypothyroidism. This study again has the strength of being the first to document thyroid function among Ugandan children and adolescents with T1DM. The 7.3% prevalence of thyroid dysfunction is similar to that reported in some studies from the United States, Germany and Turkey [4, 6, 20] but lower than that reported in other studies from Brazil and Greece [8, 18]. With regards to the type of thyroid dysfunction, findings from our study are in agreement with multiple other studies from European, North and South American countries [4, 6, 8, 11, 20]. These studies reported that the majority of subjects with thyroid dysfunction were hypothyroid with subclinical hypothyroidism, which findings are similar to those from our study.

Furthermore, subclinical hypothyroidism has been associated with an increased risk of reduced linear growth [11, 21]. In our study, we found that three out of five of the subjects with thyroid dysfunction were stunted. This notwithstanding, 33% (21/64) of the study subjects with normal thyroid function were also stunted. This is similar to what was reported in the Uganda Demographic Health Survey 2006 where 38% of all children under five years were stunted. The results in our study probably reflect a complex inter-play of other factors other than thyroid dysfunction including genetics and the environment in affecting growth and development.

Only one out of five study participants with thyroid dysfunction had concurrent thyroid autoimmunity. This is much lower that what has been reported in other studies [4, 6–9] where up to 50% of antibody positive subjects had concurrent thyroid dysfunction. This is possibly due to the non-autoimmune nature of a certain form of T1DM reported in African subjects [16]. However, it could also be due to other thyroid autoantibodies e.g. anti-thyroglobulin (antiTg) [4, 6, 7] which were not assayed in this study. However, Anti-TPO, which was assayed in this study has been shown to be more prevalent [4, 6, 15] and more specific than antiTg in predicting the development of thyroid dysfunction [6].

In addition, our study found a relationship between thyroid dysfunction and duration of diabetes mellitus with subjects with thyroid dysfunction having had diabetes for longer 4 (3.8 – 8) years vs 2.8 (1.1 – 4.4) years p = 0.049. This is similar to what was described in some studies [18] while other studies found no association [8, 15]. However, this study was not powered and the age ranges of the study subjects were too narrow to prove this as an association. This finding though does lend credence to the need for continued monitoring of thyroid function in this particular group of children and adolescents.

Furthermore, this study found a prevalence of 85.5% of poor glycaemic control indicating that almost 9 out of every 10 children are poorly controlled. This was much higher than that found in a previous but unpublished study in the same population which concluded that this was likely due to poor clinic attendance, missed insulin doses and poor insulin storage conditions. With more regular insulin supplies and the provision of an HbA1C machine donated through the International Diabetes Federation, it is expected that, coupled with ongoing diabetes education, glycaemic control in this population will improve.

This study was not without limitations, including the small sample size which was as a result of the relatively small paediatric diabetes population attending the clinic. It would have been desirable to assay samples for other thyroid autoantibodies but this was hampered by the unavailability of such tests in Uganda where the study was done. Further research would be required in the form of longitudinal follow up studies to evaluate the progress of thyroid dysfunction in this group of patients and to evaluate whether treatment of the subclinical hypothyroidism would improve diabetes control.

## Conclusion

In conclusion, these data show that thyroid autoimmunity and dysfunction are present in this group of diabetic children and strengthen the argument for routine screening of all diabetic children and adolescents for thyroid autoimmunity (particularly anti-TPO) as recommended by consensus guidelines. This is important because of the adverse effects of undiagnosed subclinical thyroid dysfunction on diabetes control, growth and development.
